# Study on Mechanical Properties and Hydration Characteristics of Bauxite-GGBFS Alkali-Activated Materials, Based on Composite Alkali Activator and Response Surface Method

**DOI:** 10.3390/ma18071466

**Published:** 2025-03-26

**Authors:** Lilong Wang, Hongkai Chen, Yannian Zhang

**Affiliations:** 1 School of Civil Engineering, Shenyang Jianzhu University, Shenyang 110168, China; 2 School of Geographical Sciences, China West Normal University, Nanchong 637002, China; 622220090029@mails.cqjtu.edu.cn; 3 School of Civil Engineering, Dalian Jiaotong University, Dalian 116028, China

**Keywords:** bauxite tailings powder, alkali-activated, compressive strength, hydration products

## Abstract

To address the limitations in determining the amount of activator and optimizing the mix proportion during the preparation of bauxite tailings (BX)-based alkali-activated materials (AAMs), as well as the insufficient research on the interactions of multiple factors, this study aims to synthesize and optimize composite cementitious materials with BX and granulated blast furnace slag (GGBFS) as precursors via response surface methodology and central composite design (RSM-CCD). The optimal alkali activator proportion and slag content for alkali-activated, bauxite tailing, powder slag cementitious materials were investigated. A series of tests, including XRD, FTIR, TG-DSC, and SEM–EDS, were used for analysis to further investigate the effects of the alkali activator dosage on the mechanical properties and the influence of the slag content on the hydration products and microstructure. The results show that the optimal composition of alkali-activated bauxite tailings-based cementitious material is 35% slag content, 4% alkali content, a water glass modulus of 1.3, and a water–solid ratio of 0.32. The relationship model between the activator parameters and compressive strength fits well, with model determination coefficients of 0.9803 for *f*_3c_ and 0.9789 for *f*_28c_. The identified hydration products were mainly C-S-H and C-(N)-A-S-H gels in the form of SiQ_3_ and SiQ_4_ tetrahedra. The SEM–EDS results show that the incorporation of slag changes the silicon–aluminum ratio of the system, promoting an increase in the content of hydration products and increasing the complexity and density of the structure. GGBFS also has a micro-aggregate filling effect and a nucleation effect, which improve the distribution of hydration products. This study demonstrates the significant potential of BX in the preparation of cementitious materials, which contributes to the sustainable development of the construction industry.

## 1. Introduction

### 1.1. Research Background

Climate change and environmental degradation have intensified the need for sustainable construction materials, as the cement industry contributes significantly to global CO_2_ emissions [1,2]. Alkali-activated materials (AAMs) have emerged as promising alternatives to ordinary Portland cement (OPC), offering substantial reductions in carbon emissions and excellent durability and mechanical properties [3,4,5]. Consequently, the development of alkali-activated materials (AAMs) has been positively impacted by the exploration of diverse precursors rich in aluminosilicates. Therefore, the development of AAMs derived from various silica-aluminate-rich precursors plays a positive role in the advancement of AAM technology.

BX, which is rich in aluminosilicate minerals such as kaolinite, is an abundant industrial by-product generated during alumina production processes, such as beneficiation–Bayer and beneficiation–sintering [6,7]. In China alone, approximately 700,000 tons of BX is produced annually [8], and most of it is stockpiled without further treatment, leading to significant environmental pollution and land use concerns [9,10]. Previous studies have explored the use of BX in concrete as an aggregate replacement and cement admixture [11,12,13,14], demonstrating its potential in construction applications. Given that BX contains substantial amounts of SiO_2_, Al_2_O_3_, and Fe_2_O_3_, which are key components for producing alkali-activated materials (AAMs) [3,8], the use of BX as a precursor in AAMs is a promising solution for waste management and sustainable material development.

### 1.2. Literature Review

#### 1.2.1. Exploration of Hydration Activation Enhancement Methods

Despite the potential of BX to produce alkali-activated materials, its primary mineral components, diaspore, kaolinite, and illite, have stable crystal structures at ambient temperatures, resulting in low geopolymer reactivity [15,16]. Therefore, enhancing the reactivity of BX is crucial. Various methods have been explored for this purpose, such as mechanical grinding and calcination. Tchadjie et al. [6] demonstrated that mechanical grinding increased the 28-day compressive strength of BX-based geopolymers by 47%, although excessive fineness led to high flowability. Calcination at high temperatures, as studied by Boum et al. [17], transformed low-activity minerals into more reactive phases, such as meta-kaolinite and hydrated corundum, increasing the compressive strength by 92% compared with that of uncalcined BX geopolymers. However, this energy-intensive process may produce additional mullite crystals that affect the hydration activity [10,17]. Increasing the alkali activator content can also increase the reactivity by promoting the dissolution of Al and Si in the precursor material. However, a high Na_2_O content can lead to excessive exothermic reactions, negatively impacting strength development during curing [9,18]. Moreover, geopolymers made solely from calcined BX under alkaline conditions often do not harden at room temperature [10]. These challenges indicate that the use of physical methods to increase BX activity is limited. Therefore, the development of combined and innovative approaches is essential for the effective application of BX in sustainable building materials.

To overcome the drawbacks of activating precursors via a single method, an increasing number of researchers are focusing on improving the overall reactivity from the perspective of the physical phase composition of the precursors [3,6]. Konduru et al. [9] prepared an aluminosilicate geopolymer using bauxite residue (BX) as the precursor and silica fume as the SiO_2_ additive. A substantial amount of reactive Si provided by the silica fume interacted with the reactive Al, promoting the formation of gel-like hydration products. Similarly, Djobo et al. [16] synthesized a ternary geopolymer using volcanic ash, aluminosilicate, and oyster shells. They reported that increasing the SiO_2_/Al_2_O_3_ and CaO/SiO_2_ ratios in the system increased the depolymerization rate of the precursors and increased the amount of hydration products, thereby improving the strength. Ye et al. [19] utilized aluminosilicate as a precursor and slag as a reinforcement, achieving a 28-day compressive strength of 50.0 MPa under 1.8 M sodium silicate activation conditions. They reported that granulated blast furnace slag (GGBFS) released a significant number of Si-O-Si bonds, promoting the formation of C-A-S-H and N-A-S-H gels. These studies indicate that while BX has relatively low reactivity, its hydration activity can be further reduced through physical activation and by combining it with multi-phase materials to achieve mutual enhancement. Therefore, by carefully selecting the appropriate precursor ratios and activation methods, alkali-activated materials (AAMs) that meet the performance requirements can be prepared.

Despite these encouraging findings, previous research on BX-based alkali-activated materials (AAMs) has focused primarily on specific aspects, such as the effects of individual precursors or single activator types on performance. Key areas, including the optimization of activator parameters, evolution of microstructure, and synergistic effects in multi-component systems, have not been thoroughly explored. Without comprehensive and systematic studies of these factors, the full potential of BX in AAMs cannot be realized. Therefore, it is crucial to conduct systematic investigations of various variables and their interactions to analyze BX-based AAMs by exploring different precursor combinations, activator types and concentrations, and curing conditions.

#### 1.2.2. Multivariate Analysis

Notably, the performance of AAMs is highly susceptible to various factors. The type and content of alkaline activators, as well as the composition of the precursor materials, are key determinants of the reaction rate, hydration product composition, microstructure, and mechanical properties [20]. Although the effect and optimal dosage of the activator are closely related to the mineral composition of the raw materials used, sodium silicate-based composite activators are generally considered the most effective compared with other types of activators (such as NaOH, Na_2_CO_3_, Na_2_SO_3_, and CaO). However, the effects of sodium silicate composite activators can be influenced by factors such as the sodium silicate modulus and water-to-solid ratio. Previous studies on these factors have often employed univariate approaches, changing only one factor at a time, such as discussing only the influence of the alkali content or the water-to-solid ratio. This limits the comprehensive understanding of the complex interactions between multiple variables in the polymerization reaction, which, in turn, hinders the comprehensive optimization of material performance. Therefore, in-depth exploration of multi-component systems, composite activator ratios, and their synergistic effects remains a critical issue in the field.

Traditional orthogonal experimental methods are commonly used to analyze the factors that influence the performance of AAMs. These methods can establish the effects of multiple factors with relatively little experimental data, thereby revealing the optimal combinations at different factor levels. However, as the number of experimental variables increases, particularly when handling multi-factor and multi-level experimental designs, the analysis becomes more complex. This is particularly true for discrete data analysis, where orthogonal methods may have limitations in accuracy and predictive ability [21]. To overcome these limitations, response surface methodology (RSM), an effective multivariate statistical optimization tool, offers new insights for the optimization of material performance. RSM builds mathematical models that not only predict the interactions between various factors with precision, but also optimize experimental designs in the presence of multiple factors, addressing the shortcomings of orthogonal experimental analysis for multi-factor studies [22]. Research has demonstrated that RSM has been successfully applied to the experimental optimization of ultra-high-performance concrete (UHPC) and cementitious admixtures [23,24]. However, few studies have applied RSM to AAMs, particularly in the preparation of bauxite residue-based AAMs (BX-based AAMs) and in exploring the interactions between factors such as the alkali equivalent, sodium silicate modulus, and water-to-solid ratio in sodium silicate composite activators. These factors may influence the material properties either individually or in pairs, and without thorough exploration, they may be insufficient to accurately predict the material performance.

This study aims to synthesize and optimize a composite cementitious material using bauxite (BX) and ground granulated blast furnace slag (GGBFS) as precursors, addressing the identified lack of systematic investigations into bauxite-based alkali-activated materials (AAMs). A composite alkaline activator of sodium silicate (Na_2_SiO_3_) and sodium hydroxide (NaOH) was employed. Using response surface methodology with a central composite design (RSM-CCD), the mix proportions of the alkali-activated BX-GGBFS composite (AABG) were designed and optimized. The macroscopic properties and workability of the AABG were comprehensively evaluated through compressive strength testing, fluidity assessments, and setting time measurements. To gain insight into the microstructural and hydration characteristics, analyses were conducted via X-ray diffraction (XRD), energy-dispersive X-ray spectroscopy (EDS), Fourier transform infrared spectroscopy (FTIR), thermogravimetric analysis, differential scanning calorimetry (TG-DSC), and scanning electron microscopy (SEM). The outcomes of this research are expected to provide a theoretical foundation for the high-value utilization of bauxite residue and promote the advancement of alkali-activated materials. By elucidating the relationships among the composition, processing parameters, and material properties, this study contributes to the development of sustainable construction materials and addresses the critical need for systematic exploration in this field.

## 2. Materials and Methods

### 2.1. Materials

The experimental materials used to develop the alkali-activated materials (AAMs) were BX, ground granulated blast furnace slag (GGBFS, S105 grade), and water. The composite alkali activators Na_2_SiO_3_ and NaOH were used. BX was obtained from a metallurgical powder supplier in Hebei Province, and GGBFS was sourced from Henan Province.

**Figure 1:**
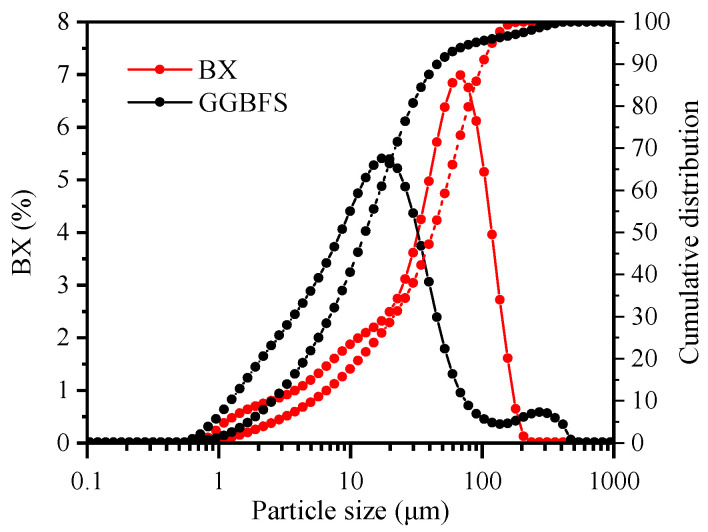
Particle size distribution of raw materials.

For preparation, both BX and GGBFS were dried at 105 °C for 24 h, ground for 15 min via a vertical planetary ball mill (XQM-4) at 3600 rpm, and, finally, sieved through a 75 µm screen. The particle size distributions of BX and GGBFS were analyzed via a laser particle sizer (Malvern Mastersizer 2000, Malvern Instruments, Malvern, UK), and the results are shown in Figure 1. The median particle sizes (D 50) of the BX and GGBFS were 42 µm and 13 µm, respectively, whereas the D90 values were 104 µm and 50 µm, respectively. The specific surface areas measured via the BET method were 3244 m^2^/kg for BX and 2525 m^2^/kg for GGBFS, indicating that both materials were ultrafine powders. Research has shown that mechanical grinding can alter the particle size distribution and increase the specific surface area. This increase in the specific surface area enhances the contact area between the alkaline solution and precursor particles, potentially improving the material reactivity. Consequently, the mechanical grinding process facilitates the breaking of Si-O-T bonds (where T represents Si or Al) within the precursor materials, thereby releasing more Al^3+^ and Si^4+^ ions [25,26]. This increased ion availability enhances the polymerization reactivity of the material [27,28].

**Figure 2:**
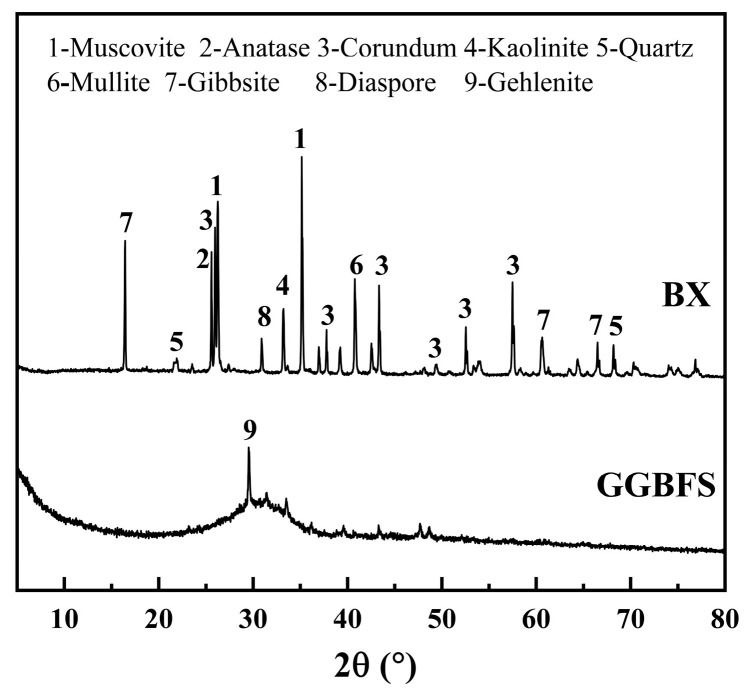
Mineral phase composition of raw materials.

The mineralogical constituents of both BX and GGBFS were established via X-ray diffraction analysis performed with a Bruker D8 Advance diffractometer (Bruker, Berlin, Germany). The results, shown in Figure 2, indicate that GGBFS is predominantly composed of gypsum, calcium silicate, and calcite, and a distinct broad hump in the 2θ range of 25° to 35° in the GGBFS diffractogram indicates a significant amount of highly reactive amorphous silica-alumina minerals [29]. The mineralogy of BX primarily consists of crystalline phases, including muscovite (PDF: 02-0055), gibbsite (Al(OH)_3_, PDF: 33-180), corundum (α-Al_2_O_3_, PDF: 10-173), and diaspore (β-AlOOH, PDF: 05-335).

The chemical compositions of BX and GGBFS were analyzed via X-ray fluorescence spectroscopy (XRF, Panalytical Axios, Almelo, The Netherlands), and the results are presented in Table 1. The analysis indicated that the GGBFS contained a relatively high CaO content of 50.22%, a basicity coefficient of 1.47, and an activity index of 0.47. These characteristics suggest that GGBFS is a highly reactive alkaline slag [30]. Bauxite (BX) primarily consisted of Al_2_O_3_ (69.234%) and SiO_2_ (21.1%). The high loss on ignition (LOI) value of bauxite implies the presence of a considerable amount of hydroxyl-bearing minerals [7,10]. The SiO_2_/Al_2_O_3_ molar ratios of GGBFS and bauxite were 2.2 and 0.3, respectively.

The alkali activator solution was prepared using sodium hydroxide, sodium silicate, and water. The amount of alkali activator is expressed in terms of Na_2_O equivalents (the mass ratio of Na_2_O to the solid precursor). Sodium silicate, produced by the Yixiang factory in Henan Province, is a fast-dissolving powder with a modulus (Ms = SiO_2_/Na_2_O molar ratio) of 2.3 (55.5% SiO_2_ and 23.2% Na_2_O). Sodium hydroxide, provided by Tianjin Zhiyuan Chemical Reagent Co. (Tianjin, China), was a 96% pure solid granule. The alkaline activator used in this study was a “modified sodium silicate solution” obtained by combining solid sodium hydroxide, sodium silicate, and water. A smaller modulus of sodium silicate corresponds to a higher concentration of OH^−^ ions in solution, resulting in a more basic substance [31,32]. To dissipate the heat released during the dissolution of sodium hydroxide, the alkaline activator was prepared 24 h prior to testing. Although the effectiveness of activators is closely related to the mineral composition of the raw material used [33], compared with other types (NaOH, Na_2_CO_3_, Na_2_SO_4_, and CaO), sodium silicate is considered one of the most effective activators, because it can supply active silicon during initial hydration [34,35].

### 2.2. Response Surface Experimental Method

Response surface methodology (RSM) was employed for optimization to systematically investigate the influence of the alkali content, sodium silicate modulus, and water-to-solid ratio on the strength development of alkali-activated bauxite-GGBFS (AABG) composite materials, and response surface methodology (RSM) was employed for optimization. These parameters are known to directly affect the engineering performance of cementitious materials; therefore, their optimization is of paramount importance [6,9]. Through the application of RSM, the interactions among these parameters can be effectively analyzed, facilitating the determination of optimal mix proportions that maximize both the mechanical properties and hydration characteristics of the material. The specific procedure involved the following steps: (1) the alkali content, sodium silicate modulus, and water–solid ratio (W/B, defined as the ratio of free water to the total weight of the precursor within the cementitious material) were designated as independent variables. On the basis of prior research, approximate parameter ranges were established, and a central composite design (CCD) was implemented for the experimental design. (2) Subsequently, a response surface model was constructed by analyzing the experimental data. This model was used to predict the optimal range of the mixture proportions. (3) Experimental validation was performed using selected optimal mix proportions. By comparing the performance with previous studies and observing the workability during the actual process, the final range and optimal mix proportion were determined.

On the basis of previous work [6,19], when m(BX)/m(GGBFS) = 65:35, the water–solid ratio varies from 0.29 to 0.35, the alkali content varies from 1% to 7%, and the modulus of sodium silicate varies from 1.0 to 1.6.

The experimental results were analyzed via the software “Design Expert 10.0”, and the effects between the factors of the experimental variables were investigated via the response surface methodology and central composite design (RSM-CCD) method. The coding and horizontal design of the argument variables are listed in Table 2. To thoroughly evaluate the performance attributes of the cementing material and refine its mix design, the 3-day compressive strength (*f*_3c_) and 28-day compressive strength (*f*_28c_) were selected as the key response parameters. A three-factor and five-level experimental configuration was designed, and 20 experiments were designed, among which 14 groups were factorial points and six were central point repeated verification groups. The matching schemes are listed in Table 3. The determination coefficient (*R*^2^), coefficient of variation (*C.V*), and adjusted coefficient of determination (*R*^2^_adj_) were used as the key parameters for analysis of variance (ANOVA) and model accuracy judgment.

### 2.3. Sample Preparation

**Figure 3:**
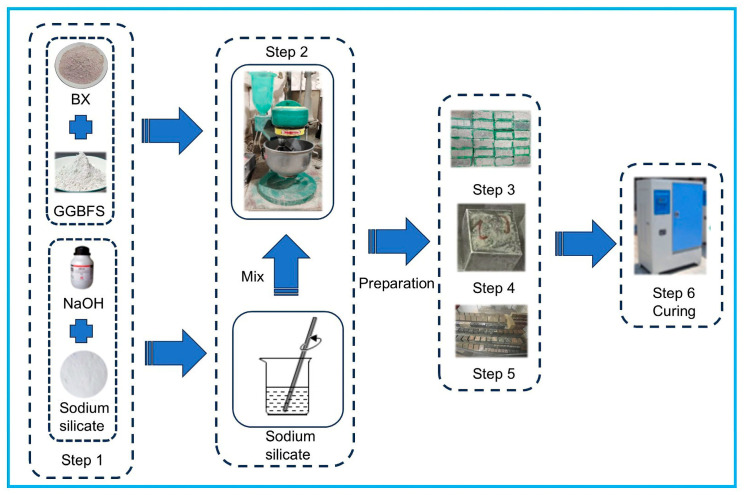
Specimen preparation process.

Figure 3 illustrates the sample preparation procedure. Before mixing, BX and GGBFS were dried. The precursor materials were weighed according to the mass percentages listed in Table 3, and mixed thoroughly for 30 s in a JJ-5 planetary mixer to ensure uniform dispersion. The alkali activator solution was slowly added to the raw material mixture and stirred rapidly for 60 s to homogenize the fresh geopolymer paste. The mixture was allowed to stand for 90 s, and the paste was scraped from the inside of the mixing pan. The AAMs were rapidly stirred for 60 s to complete the preparation. The prepared slurry was poured into 40 × 40 × 40 mm triple molds. After being cast, the samples were vibrated on a shaking table for 60 s to eliminate entrapped air bubbles, and then covered with a polyethylene film. The mold was placed in a standard curing room (20 ± 2 °C; RH ≥ 95%). The mold was released after curing for 24 h, and the sample was cured in a standard curing box for different durations (3 d and 28 d).

### 2.4. Test Method

After the samples were prepared and cured according to the conditions outlined in Section 2.3, their properties were evaluated via a series of tests. The subsequent sections describe the experimental methods used to assess the workability, mechanical properties, and microstructural characteristics of the alkali-activated materials (AAMs). These evaluations provide insights into the performance of the samples and the effects of different mix proportions prepared via response surface methodology (RSM). Further investigations will assess the effects of the GGBFS content on both the workability and mechanical properties.

#### 2.4.1. Workability Analysis Method

The fluidity diameter of the fresh slurry was evaluated according to the skip test method specified in the Chinese standard GB/T 2419-2005 [36]. The average of three test results was taken for each group, to characterize the mobility of the group. The test was carried out after the preparation of the sample. A caliper was used to measure the maximum spreading diameter of the bottom surface of the slurry, and the average value was recorded as the flow value.

#### 2.4.2. Mechanical Performance Analysis Method

The compressive strength was evaluated in accordance with the Chinese GB/T 17671-1999 standard [37]. Using a GYE-300B universal testing machine, the samples underwent compressive testing at a consistent loading rate of 2.4 kN/s. Strength data were recorded on days 1, 3, 7, and 28. To represent the compressive strength of each group, mean values derived from triplicate measurements at each time interval were employed.

#### 2.4.3. Analysis of Hydration Products

The hardened paste samples were crushed and immersed in anhydrous ethanol for 24 h to stop the hydration process. Before the reaction product analysis, the paste sample was ground for 10 min with an agate mortar and passed through a 75 mm sieve.

(1) A Bruker D8 Advance X-ray diffractometer (XRD) from Germany was used for qualitative crystal structure analysis of the AABG products, employing CuKα radiation (40 mA, 40 kV). XRD patterns acquired from 5° to 70° at a scan rate of 5°/min revealed that crystalline phases, such as calcium silicate hydrate (C-S-H), formed during hydration. This information aids in understanding the reactivity of BX and GGBFS under alkali activation and their impact on mechanical performance.

(2) The material functional groups and elemental bond energies of the AABG were analyzed via FTIR (Thermo Scientific Nicolet iS50, Madison, WI, USA) within the wavenumber range of 400–4000 cm^−1^. FTIR analysis allows for the identification of alterations in the Si-O-T (T = Si or Al) bonds within the hydration products. Furthermore, it enables the analysis of chemical bond breakage and reorganization during polymerization reactions, thereby providing insights into the mechanisms of polymerization reactivity and material mechanical property enhancement.

(3) A German Netzsch STA449 F5 synchronous thermal analyzer (TG-DSC, Netzsch STA449 F5, Netzsch, Selb, Germany) was used to analyze the type and degree of hydration products, which were heated in a nitrogen test atmosphere within the range of 30–1000 °C (heating rate 10 °C/min). Heat insulation is not required during the heating process. TG–DSC analysis enables the determination of the thermal decomposition temperature and mass loss of hydration products, and the assessment of the type and content of the hydration products. This aids in understanding the thermodynamic properties of hydration reactions and their contributions to the mechanical properties of materials.

#### 2.4.4. Microstructural Analysis of Hydration Products

The AABG hydration product composition and morphology were characterized via a ZEISS Gemini 300 field-emission electron microscope (SEM + EDS, ZEISS GeminiSEM 300, Jena, Germany). SEM images were acquired at magnifications of 2K×, 10K×, 20K×, and 60K×, with an acceleration voltage of 10 kV and a working distance of 5–6 mm. Prior to SEM–EDS analysis, the samples were coated to enhance signal detection. This SEM–EDS analysis enabled observation of the hydration product microstructure and morphology, identification of different hydration product distributions and morphologies, and quantitative elemental distribution analysis. This information is crucial for understanding the hydration reaction mechanism and its effect on the mechanical properties of a material.

## 3. Results and Analysis

### 3.1. Response Surface Modeling and Optimization

#### 3.1.1. Modeling Regression

The strengths of the 20 samples obtained by the RSM-CCD experiment are listed in Table 4. A mathematical model of the regression between the 3-day compressive strength (*f*_3c_) and 28-day compressive strength (*f*_28c_) and the independent variables *X*_1_, *X*_2_, and *X*_3_ was fitted via the least squares method, as expressed in the following formula:

(1) $$f_{3c}=53.44-3.94X_{1}+15X_{2}-8.19X_{3}-2.26X_{1}X_{2}+0.93X_{1}X_{3}+4.59X_{2}X_{3}-0.96X_{1}^{2}-0.908X_{2}^{2}+0.96X_{3}^{2}$$

(2) $$f_{28c}=82.71-1.37X_{1}+18.36X_{2}-8.15X_{3}-1.67X_{1}X_{2}+2.11X_{1}X_{3}+6.42X_{2}X_{3}-0.7X_{1}^{2}-10.27X_{2}^{2}+2.02X_{3}^{2}$$

#### 3.1.2. Model Applicability and Significance Analysis

The applicability of the developed response surface models was evaluated via analysis of variance (ANOVA), and the results are summarized in Table 5. The coefficient of determination (*R*^2^), coefficient of variation (*C.V*), and adjusted *R*^2^ (*R*^2^_adj_) were used to assess the overall accuracy and goodness-of-fit of the models. A higher *R*^2^ value indicates a greater proportion of variance explained by the model, suggesting enhanced predictive capability [38]. The *R*^2^ values of the *f*_3c_ and *f*_28c_ models were 0.9803 and 0.9789, respectively. This meant that there was only 1.97% and 2.11% of variation that the *f*_3c_ and *f*_28c_ models could not explain, respectively. The *C.V* represents the degree of fit between the regression model and sample data points, defined as the ratio of the standard error of the estimate to the mean observed response, and provides a measure of the model’s precision and reliability. A coefficient of variation (*C.V*) of less than 10% was observed for both the *f*_3c_ and *f*_28c_ models. This low *C.V* indicates that the models accurately represent the experimental data with strong stability and reproducibility [39,40], while also implying a minimal level of unexplained variability or noise in the data relative to the magnitude of the responses being modeled.

ANOVA provides *p*_values_ and *F*_values_ that allow researchers to determine the significance of individual independent variables and the extent to which interaction effects among independent variables affect the response variable [41]. *p*_Value_ describes the correlation between the model-independent variable and the experimental data. Typically, when *p*_Value_ < 0.05, the model variable significantly affects the intensity. The magnitude of *F*_Value_ provides a measure of the extent to which each factor contributes to the overall variation in the response. The greater the *F*_value_, the greater the degree of influence of this factor. In the *f*_3c_ model, the values of the *X*_1_, *X*_2_, and *X*_3_ factors are 0.0013, <0.0001, and <0.0001, respectively, and the *p*_Value_ of the *f*_28c_ model are 0.2262, <0.0001, and <0.0001, respectively, all of which are less than 0.05. Therefore, *X*_1_ and *X*_3_ are significant independent variables affecting the compressive strength, and *X*_1_*X*_2_ and *X*_2_*X*_3_ are significant interaction terms. The alkali content (*X*_1_) and water–solid ratio (*X*_3_) significantly affect the compressive strength.

**Figure 4:**
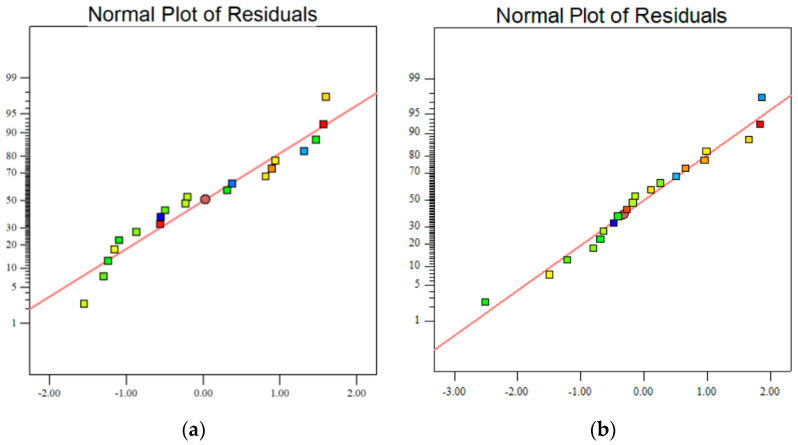
Intensity data distribution map: (**a**) *f*_3c_ and (**b**) *f*_28c_.

A normal probability graph can further describe the rationality of the data distribution and model establishment [42]. As depicted in Figure 4, the near-linear distribution of the data points for all the dependent variables indicates that the residuals are normally distributed. The smooth fit of the data to the model-predicted lines provides strong evidence that the model effectively captures the relationship between the independent and dependent variables. This validation confirms the suitability of the model for analyzing the effects of each factor on the compressive strength.

#### 3.1.3. Corresponding Surface Analysis

Response surface diagrams illustrate the influence of interacting variables on the response value, with the curvature of the surface indicating the strength of the interaction [38]. The greater the curvature is, the more significant the effect.

**Figure 5:**
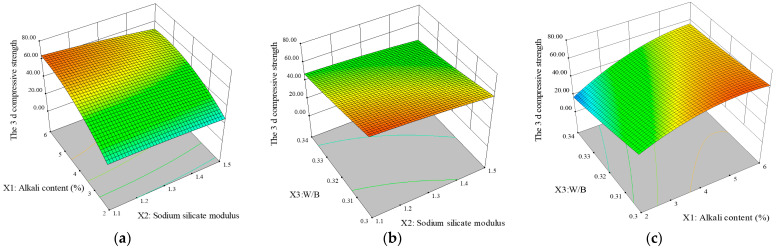
Three-dimensional response surface plots for 3 d compressive strength: (**a**) *X*_1_*X*_2_ factor, (**b**) *X*_3_*X*_2_ factor, and (**c**) *X*_3_*X*_1_ factor.

Figure 5 shows the relationships between different factors and the response variable *f*_3c_ via a three-dimensional response surface. The response surface presents a three-dimensional paraboloid with an upward opening, indicating that factors such as the alkali content, sodium silicate modulus, and water–solid ratio significantly affect the compressive strength. Figure 5a shows that *f*_3c_ tends to increase with increasing Na_2_O equivalent. When the Na_2_O equivalent is unchanged, the sodium silicate modulus is inversely proportional to the compressive strength; when the Na_2_O equivalent is unchanged, the sodium silicate modulus is inversely proportional to the compressive strength. Figure 5b shows that the fixed water–solid ratio *X*_3_ and 3 d compressive strength *f*_3c_ decrease with increasing sodium silicate content *X*_2_. However, regardless of the amount of water glass used, the strength increases as the value of *W*/*B* decreases, and *f*_3c_ reaches its maximum value when the water–solid ratio *X*_3_ is 0.3 to 0.32.

Figure 5c shows the interactive effects of the W/B ratio and alkali equivalent on the compressive strength. When the activator content is constant, increasing the W/B ratio leads to a modest reduction in strength. However, the alkali equivalent displays a more complex relationship: the strength increases to a peak, and then decreases as the alkali equivalent increases, independently of the W/B ratio. These findings indicate that the impact of the alkali content on the AABG compressive strength outweighs the impact of the W/B ratio. An alkali content that is too low or too high prevents the development of AABG strength [9]. When the alkali content is low, fewer Si^4+^ ions are released by dissolution, and the amount of GGBFS involved in the cementation reaction is limited. This makes it difficult to form a dense microstructure with relatively high strength [43]. The elevated pH of the alkaline solution drives the polycondensation reaction, leading to the dissolution of the [SiO_4_]^−^ and [AlO_4_]^5−^ structures and the subsequent formation of C-A-S-H and N-A-S-H gels. These gels are crucial for improving the compressive strength of materials. However, when the alkali content is excessive, the dissolution rate of precursor particles is accelerated, and a substantial amount of gel is wrapped around the precursor particles; this has the effect of inhibiting the dissolution of the precursor particles, and is not conducive to the formation of the hydration product [18]. High alkali concentrations increase free water consumption, hinder ion migration, and diminish the gel polymerization process, reducing the number of gels and decreasing the compressive strength. Because water glass is modified with NaOH, a higher water glass modulus corresponds to a lower NaOH content and, consequently, lower alkalinity, resulting in reduced strength [31]. Therefore, the ideal base content of *f*_3c_ is 4% to 6%, the ideal modulus of sodium silicate is 1.1 to 1.3, and the ideal W/B range is 0.3 to 0.32.

**Figure 6:**
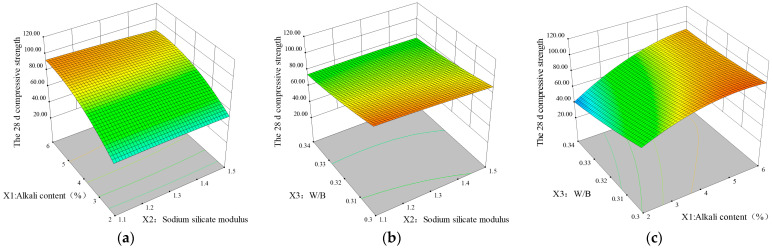
Three-dimensional response surface plots for 28 d compressive strength: (**a**) *X*_1_*X*_2_ factor, (**b**) *X*_3_*X*_2_ factor, and (**c**) *X*_3_*X*_1_ factor.

The three-dimensional response surface of *f*_28c_ is shown in Figure 6, and all the paraboloids with openings are up or down. The overall trend is similar to the three-dimensional surface diagram of *f*_28c_. In contrast to *f*_3c_, the modulus has a minimal effect on *f*_28c_, as illustrated in Figure 6a,b. As shown in Figure 6a, the maximum values of the fixed alkali content *X*_1_ and *f*_28c_ appear in the range of water glass moduli of 1.1~1.2. When the sodium silicate modulus (*X*_3_) is held constant, the highest 28-day compressive strength is observed when the alkali content (*X*_1_) is within the range of 5–6%. Furthermore, the analysis of Figure 6b,c reveals that the maximum value of *f*_28c_ is attained when the water–solid ratio (*X*_2_) is between 0.3 and 0.31.

The influence of each factor on the 3-day and 28-day compressive strengths follows the order following: alkali content of activator (*X*_1_) > water–solid ratio (*X*_3_) > modulus of sodium silicate (*X*_2_). Furthermore, the interaction term between the alkali content and the W/S ratio (*X*_1_*X*_3_) has the greatest impact, which aligns with the *p* value results in Table 5. Therefore, this section focuses on identifying the crucial independent variables and their interactions that determine the behavior of cementitious materials, setting the stage for defining the appropriate parameter conditions in the following analysis.

#### 3.1.4. Model Validation and Optimization Results

**Figure 7:**
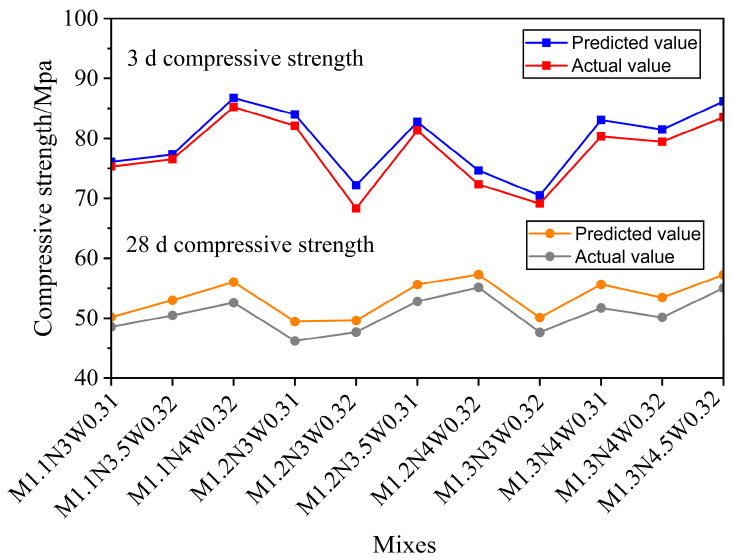
Predicted versus actual measurements.

On the basis of the optimal mixing ratio determined via response surface analysis, we further investigated the mechanical properties of the materials to verify the validity of the optimization. On the basis of the findings from the previous section regarding the effects of the alkali content (*X*_1_), sodium silicate modulus (*X*_2_), and water–solid ratio (*X*_3_) on the response values of the composite cementitious materials, Design Expert software (Design-Expert 10.0) was utilized to determine the optimal conditions for these research factors [38]. The fluidity, setting time, and compressive strength during preparation were prioritized to determine the final optimal conditions for these factors. The optimization ranges and standards used are listed in Table 6. Figure 7 compares the results of the AABG optimization scheme with the actual measured values. Experimental validation revealed that the error between the model predictions and actual measurements was within acceptable limits, indicating the high accuracy of the model. Therefore, the ideal ratio is as follows: a base content *X*_1_ of 4%, a sodium silicate modulus *X*_2_ of 1.3, and a water–solid ratio *X*_3_ of 0.32. We successfully determined the optimal mixture ratio through response surface methodology optimization, which directly fulfilled our research goal of optimizing the properties of BX-GGBFS alkali-activated materials.

### 3.2. Macroscopic Performance Analysis

#### 3.2.1. Mechanical Properties

**Figure 8:**
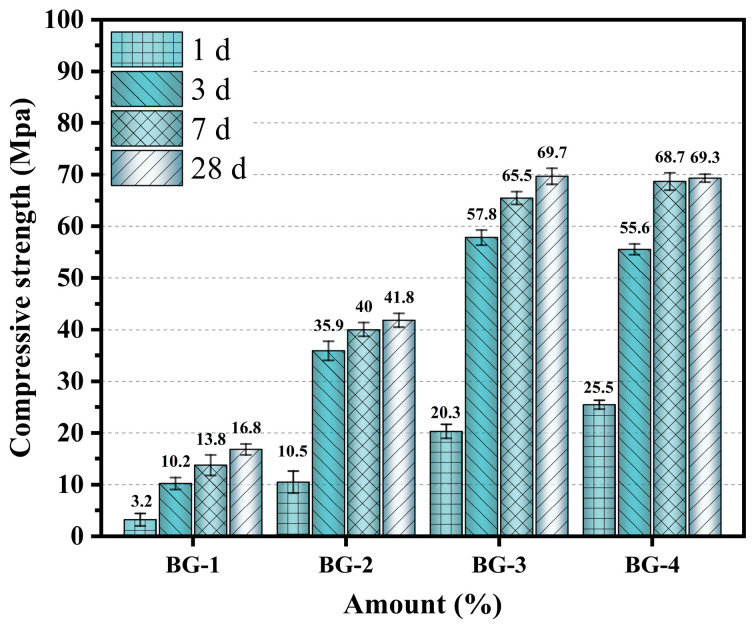
Compressive strength of AABG at 1, 3, 7, and 28 d.

Figure 8 presents the compressive strength test results for the AABG samples with varying GGBFS contents at the ages of 1, 3, 7, and 28 days. With increasing GGBFS content, the compressive strength of the AABG increased continuously, but the optimization effects for different ages were different. An increase in the GGBFS content significantly increased the 3 d compressive strength, especially when the GGBFS content was 35%, allowing it to reach 57.8 MPa. This is important for engineering applications that require rapid hardening, and fills a gap in research on the rapid construction of BX-based materials [44,45]. In general, GGBFS has a pronounced beneficial effect on improving the mechanical properties at early ages. Concurrently, the Al-rich BX contributed to the formation of N-A-S-H, whereas excess Al reacted with a portion of the C-S-H gels to produce mixed C(N)-A-S-H gels, leading to a rapid increase in strength between 3 and 7 days.

When the compressive strength reached 69.7 MPa at 28 days, the hardened slurries far surpassed the European Union’s minimum strength requirement of 32.5 MPa for construction materials [18]. However, GGBFS was a minority fraction of the mixed powders in the BG-1 group, and reactions involving GGBFS lost their dominance, resulting in a lower strength at 28 d. When GGBFS doping levels are low, [SiO_4_]^−^ and [AlO_4_]^5−^ monomers are liberated from BX by activators, resulting in the formation of N-A-S-H gels, which constitute the primary reaction pathway [6]. With increasing GGBFS content, the hydration process generates a greater quantity of gels, including C-S-H and C-A-S-H, which fill the voids between particles, thus promoting further strength development. Moreover, the incorporation of GGBFS improved the particle size distribution of the mixture (GGBFS, D50 = 22.9 μm; BTs, D50 = 45.77 μm); the fine particles within the GGBFS material were able to occupy the spaces between larger particles, thereby refining the pore structure and enhancing the overall strength [29]. Thus, an optimal raw material ratio promotes the hydration reaction and increases the micro-aggregate filling effect [29] and the nucleation effect of the material itself [17].

#### 3.2.2. Flowability and Setting Time

**Figure 9:**
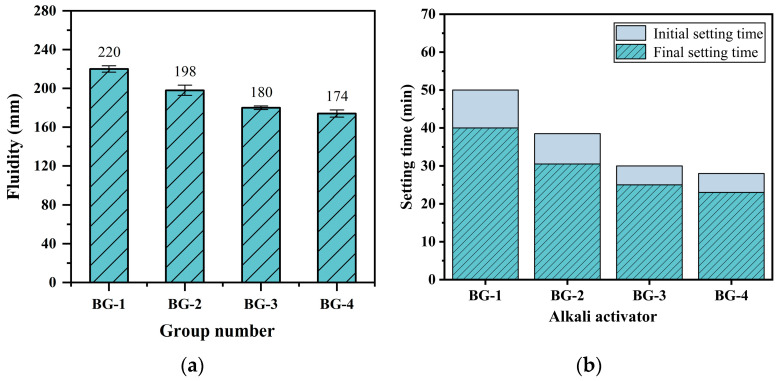
AABG with different ratios of GGBFS: (**a**) flowability and (**b**) setting time.

Figure 9a shows that the AABG fluidity at 28 days decreases with increasing slag content. At 45% slag content, the fluidity falls below the GB 175-2007 standard requirement of 180 mm slurry flow [46]. Under alkaline conditions, the active substance in GGBFS can rapidly polymerize with the active Al in the BTs, and consume free water in the system. The increased formation of hydration products within the gelling material contributes to a marked reduction in fluidity [47]. Furthermore, a higher slag content accelerates the hydration rate, resulting in a further decrease in fluidity.

Figure 9b illustrates the variation in the AABG setting time. The addition of slag significantly shortens both the initial and final setting times of the slurries. Compared with BX, GGBFS has greater hydration activity and depolymerizes more easily under the action of activators to increase the Si-O and Al-O bond concentrations required for condensation reactions. Depolymerized Ca^2+^ ions can balance the charge and promote the generation of C-(A)-S-H gels. The system undergoes a rapid condensation reaction under the influence of Ca^2+^; in other words, this is the initial coagulation of the slurry. Compared with that of BG-3, the setting time of BG-4 is reduced by only 6.67%. This is because the depolymerization process is not solely dependent on the inherent properties of the precursor. The pH of the alkaline solution also plays a role, and the alkaline environment does not universally accelerate the depolymerization of particles.

### 3.3. Hydration Product Analysis

#### 3.3.1. XRD Analysis

**Figure 10:**
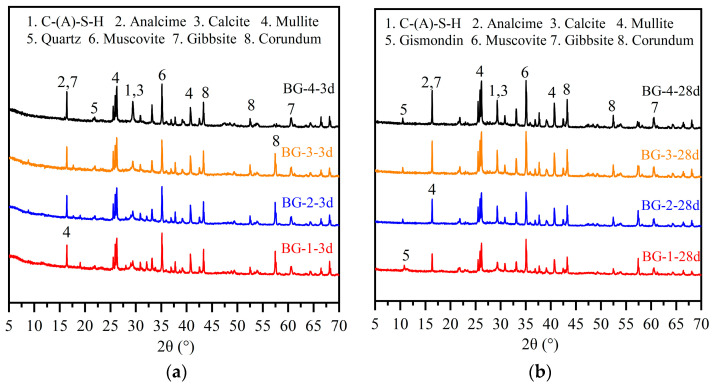
XRD analysis of AABGs with different ratios of GGBFS: (**a**) at 3 d; (**b**) at 28 d.

Figure 10 shows the XRD patterns of the AABGs with different GGBFS contents at 3 d and 28 d. Compared with the XRD results of the raw material, all the AABG samples present broad diffraction peaks at 20–40°, which are mainly related to the newly formed amorphous gels of the reaction products [48]. Despite the observation of reflections suggestive of C-(A)-S-H gel in all the samples, the semi-amorphous character of the gel and the interference from calcite (PDF: 99-0022, CaCO_3_) diffraction peaks at approximately 2θ = 29° hinders the unambiguous identification and differentiation of the C-(A)-S-H gel peaks via X-ray diffraction (XRD). Among the hydration products, analcime (PDF: 99-007, Na(AlSi_2_O_6_)) and gismondine (PDF: 00-020-0452, CaAl_2_Si_2_O_8_·4H_2_O) are also observed. Analiques and other zeolite-phase substances have also been observed in the hydration products of samples prepared by sodium silicate-excited metakaolin, which are the crystals of N-A-S-H gel formed by the coprecipitation of Na^+^, Al^4+^, and Si^3+^ under alkaline conditions (pH > 9) [49]. Research has shown that zeolite-phase substances can fill gel pore cavities and increase the strength of hardened pastes [6]. However, the identification and distinction of amorphous N-A-S-H and C-A-S-H gels are difficult. On the basis of the raw material composition, these gels were categorized as C-S-H (PDF: 33–0306) gels and C-N-A-S-H gels.

A comparison of Figure 10a,b shows that the composition of the AABG hydration products is essentially unchanged at different ages. However, the hump area in the 20–40° region expands, indicating that the amount of amorphous gel produced by polycondensations increases [29]. As the GGBFS content increases, the Ca/Si ratio in the AABG system decreases. Concurrently, Na+ ions in the system progressively replace Ca^2+^ ions, adsorbing onto the gel surface or interlayer spaces to maintain the charge balance. This process facilitates the ongoing transformation of the hydrate C-A-S-H within the gelling system into C-(N)A-S-H [50]. Furthermore, XRD analysis reveals persistent diffraction peaks corresponding to muscovite (β-AlOOH) and gibbsite (α-AlOOH), a peak at 2θ = 26.6°, and strong quartz (PDF: 78-1253, SiO_2_) diffraction peaks. This indicates that a portion of the BX remained unreacted within the AAMs, and did not participate in the polymerization. The two substances exhibited lower geopolymer reactivity, owing to their highly crystalline structures.

#### 3.3.2. FTIR Analysis

**Figure 11:**
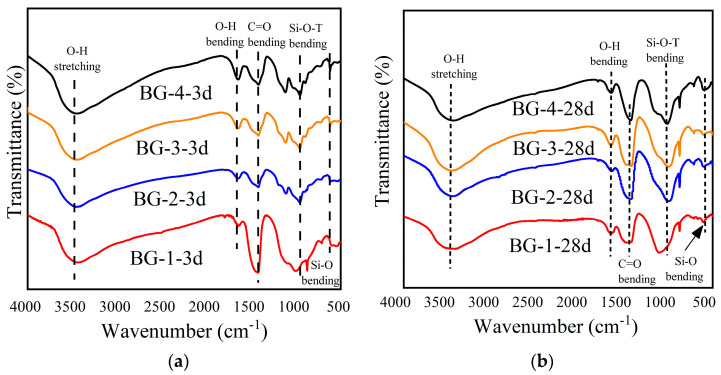
FTIR spectra of AABGs with different GGBFS contents: (**a**) at 3 d; (**b**) at 28 d.

The alterations in the hydration products of the AABG pastes with varying GGBFS contents at 7 and 28 d were characterized via FTIR to further ascertain the hydration products. The results are shown in Figure 11. The characteristic absorption peaks observed within the ranges of 1642~1654 cm^−1^ and 3442~3462 cm^−1^ are typically attributed to the stretching and bending vibrations of hydroxyl groups within water molecules [33,51]. These vibrations are primarily associated with structural water within the hydration products, such as C-S-H and C(N)A-S-H gels, as well as water adsorbed onto the precursor material [52]. The wide bands at 875 cm^−1^ and 1418 cm^−1^ were caused by asymmetrical vibrations of the O-C-O bond of the CO_3_^2−^ group. The presence of stretching/bending vibrations associated with the C-O bond suggests that carbonation occurred within the AAMs during the hydration process [53].

The primary vibrational spectral band of the hydration product gel is observed near 1000 cm^−1^, corresponding to the vibration of T-O-T bonds (where T = Si or Al) within the aluminosilicate gel structure [31,33,52]. The wavenumber of the Si-O-Al bond is generally understood to be lower than that of the Si-O-Si bond [52]. The observed shift in the characteristic peak to a lower frequency with increasing GGBFS content indicates the formation of a greater number of Si-O-Al bonds within the system. Moreover, owing to the influence of GGBFS, the structure and composition of the gel are further changed by the presence of Ca^2+^, and the N-A-S-H gel is converted into a C-A-S-H gel in a high-pH environment, ultimately leading to the coexistence of the two gels [50,54]. Therefore, the vibration spectrum band of the hydrated product is higher than that of the C-A-S-H gel (950 cm^−1^) and lower than that of the N-A-S-H gel (1020 cm^−1^). From 3 d to 28 d, the characteristic peaks continue to move toward lower waves, with narrower wideband edges and sharper peaks near 1000 cm^−1^, indicating more hydration product generation.

**Figure 12:**
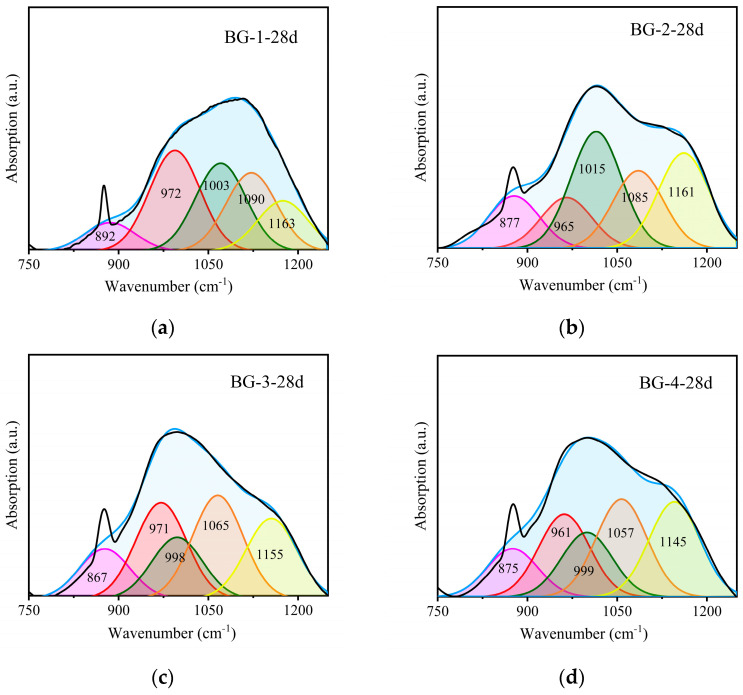
Peak fitting diagram of AABG 28 d hydration products with different GGBFS contents.

To further analyze the types of hydration products, the characteristics of the Si-O stretching vibration absorption peaks were considered in relation to the number of bridging oxygen atoms coordinated to silicon within the [SiO_4_] tetrahedron of the hydration product. The Si atoms can be categorized as follows: SiQ_0_ (near the absorption peak at 850 cm^−1^), SiQ_1_ (near the absorption peak at 950 cm^−1^), SiQ_2_ (near the absorption peak at 1000 cm^−1^), SiQ_3_ (near the absorption peak at 1050 cm^−1^), and SiQ_4_ (near the absorption peak at 1100 cm^−1^) [55,56]. Free Al^3+^ can replace Si atoms in the [SiO_4_] tetrahedra during the reaction, forming the [AlO_4_] structure. However, this process does not change the amount of ligand-bridging oxygen in the hydration product gel [18]. Therefore, the change in the amount of ligand-bridged oxygen in the gel can be utilized to reflect the degree of polymerization of the [Si(Al)O_4_] tetrahedra. As illustrated in Figure 12, the distinctive peaks of the 28 d hydration products were subjected to back-convolution [56]. The degree of polymerization of the AABG was determined by calculating the area ratio of (SiQ_3_ + SiQ_4_)/(SiQ_1_ + SiQ_2_) to assess the percentage of characteristic peaks present in the hydration products [57]; the results are shown in Table 7. The analysis revealed a direct correlation between the proportion of SiO_4_ tetrahedra, primarily SiQ_3_ and SiQ_4_ units, present within the C-S-H and C-(N)-A-S-H gel hydration products and the increasing proportion of slag in the mixture. This finding is consistent with the results obtained from the XRD analyses and macroscopic mechanical property evaluations.

#### 3.3.3. TG–DSC Analysis

**Figure 13:**
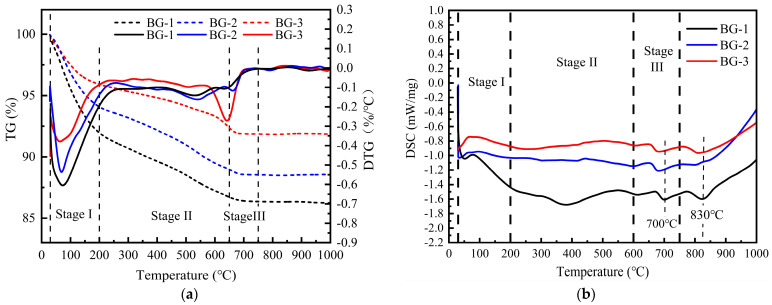
TG–DSC of AABGs with different alkali activators at 28 d: (**a**) TG; (**b**) DSC.

Figure 13 shows the TG–DSC curves of the AABG pastes cured for 28 days with varying GGBFS contents, allowing for the analysis of AABG hydration via simultaneous thermal analysis. The TG curves indicate three primary stages of mass loss: Stage I: 30–200 °C intervals for the decomposition of free, adsorbed, and weakly bound water in the C-(A)-S-H gel [18]. As shown in Table 8, the AABG losses in this interval were 4.566% (BG-3), 6.443% (BG-2), and 10.102% (BG-1), representing a higher content of gel components of the hydration products in the BG-3 system. When the polymerization reaction was insufficient, water was adsorbed on the surface of the precursor particles, instead of being hydroxylated with the gel in the form of T-OH (T = Si, Al) [19]. Consequently, the geopolymers of the BG-1 group exhibited more significant weight loss, owing to evaporation and dehydration at temperatures below 200 °C. The principal mass loss during Stage II occurred within the temperature range of 200–650 °C, a phenomenon associated with the dehydroxylation of structurally bound water within the C-(A)-S-H and N-A-S-H gels [29]. However, in TG analysis, it is often challenging to precisely differentiate between hydration products, because of the overlapping temperature ranges of their heat loss [48]. Research has demonstrated that the temperature of water loss (dehydration) in N-A-S-H gels is typically higher than that in C-A-S-H gels. Furthermore, the weight loss interval of the gels could reach 700° [58]. Structurally evolved slag-based soil aggregates, which are dominated by C-A-S-H gels, have been shown to have lower loose-bound water loss temperatures than fly ash, which is dominated by N-A-S-H gels [59]. Therefore, it can be assumed that the hydration product of the BG-3 system contained a certain amount of N-A-S-H gel. Stage III: The sustained weight loss in the temperature range of 650–750 °C is due to the thermal decomposition of carbonate [18].

DSC analysis was performed to identify the hydration products of AABG. The presence of a heat absorption peak at approximately 700 °C, indicative of calcite (CaCO_3_) decomposition [19], and an exothermic peak at 830 °C, attributed to the crystallization transformation of β-silica in the C-S-H gel [18], suggest that C-S-H is a primary hydration product. This is further supported by the observation that GGBFS promotes C-S-H gel production in the AABG system, which is consistent with the XRD and FTIR results. Therefore, the main hydration products of the AABG are C-S-H gels and C(N)-A-S-H gels.

#### 3.3.4. SEM + EDS Analysis

**Figure 14:**
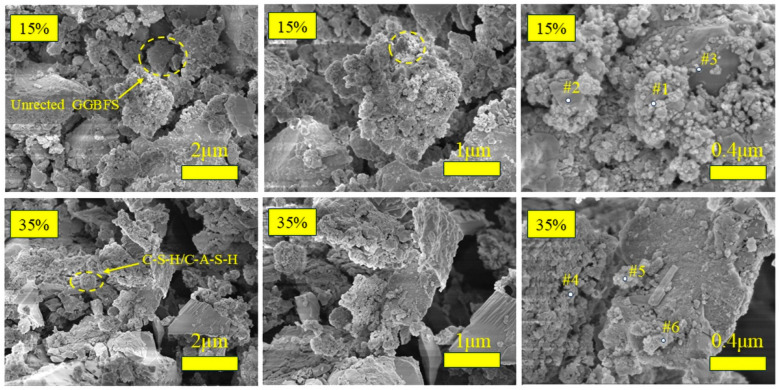
SEM images of AABG at different GGBFS levels.

Figure 14 shows SEM images of the hardened pastes from groups BG-1 (15% GGBFS) and BG-3 (35% GGBFS) after 28 days of curing. In these images, the hydration product gels appear as clustered formations, whereas unhydrated particles are visible as white regions. EDS analysis revealed that these gels were primarily composed of Ca, Si, Al, Na, and O, which is consistent with the elemental composition of the C-A-S-H gels (Table 9). Studies have shown that C-S-H, C-A-S-H, and N-A-S-H are formed simultaneously during hydration [19,29]. As the slag content increased, the calcium-to-silicon ratio (Ca/Si) in the hydrated products decreased from 0.97–1.21 to 0.7–1.0, indicating increased C-(N)-A-S-H gel formation. Simultaneously, the silicon-to-aluminum ratio (Si/Al) shifted from 0.51–1.71 to 0.64–1.27, suggesting a higher relative silicon content in the hydration products and increased Si-O-Si bond formation within the gel structure. The incorporation of GGBFS promoted the transformation of Si-O-Si bonds to Si-O-Al bonds, changing the Si/Al ratio and increasing the content and complexity of the hydration products. Therefore, optimizing the GGBFS ratio and adjusting the precursor phase composition are crucial for achieving a denser hydration product structure and a more homogeneous spatial distribution.

## 4. Discussion

### 4.1. Discussion of Hydration Mechanism

**Figure 15:**
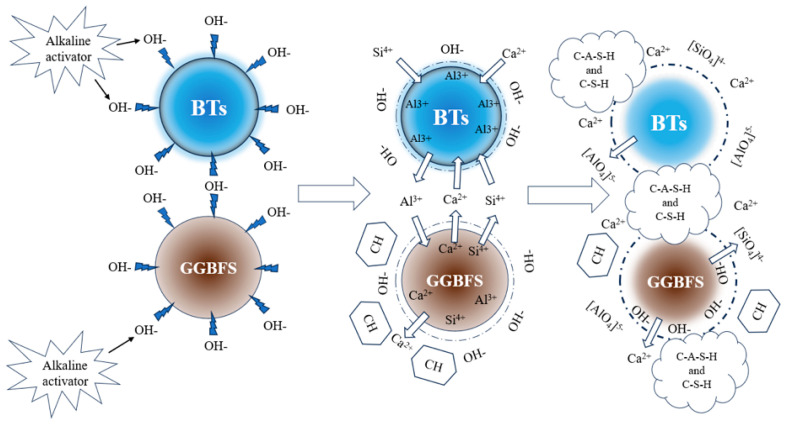
AABG formation mechanism.

Integrating the results of the analyses presented in Figure 15, the proposed mechanism by which GGBFS facilitates the hydration reaction in BX-based alkali-activated materials is outlined. This mechanism involves an increase in the calcium content due to the addition of GGBFS, which stimulates the generation of C-S-H-type gels, and, subsequently, improves the mechanical performance. In addition, the pozzolanic and micro-aggregate effects of GGBFS lead to altered hydration products, resulting in higher concentrations of both silicate and silica-aluminate gels and a denser composite cementitious matrix. With increased GGBFS doping, the hydration products are gradually converted from a C-S-H gel to a C-A-S-H gel, gradually increasing the gel content. The role of GGBFS in enhancing the microstructure of gelling materials was analyzed, and the role of GGBFS can be summarized in terms of its plasticizing effect and enhancement effect.

(1) Plasticizing effect: The addition of GGBFS optimizes the particle size distribution of the mixture (GGBFS, D50 = 22.9 μm; BTs, D50 = 45.77 μm). The GGBFS contains a certain amount of small particles, which can fill the pore space between the particles of the BTs to improve the particle gradation of the system, and the particles that fill in the gaps fill the gap between the original replacement water and free water, to promote the hydration reaction process and change the work performance.
(2) Enhancement: As a highly active silica-aluminate precursor, the active Ca component of GGBFS rapidly participates in the reaction and promotes the formation of the gel phase under the action of the activator. Alkalinity destroys the silica–oxygen protective layer on the GGBFS surface. Because [SiO_4_]^−^ and [AlO_4_]^5−^ have not yet been dissolved in large quantities, when a large amount of Ca^2+^ accumulates in the solution up to a specific concentration, Ca(OH)_2_ crystals precipitate, which are enriched around the GGBFS and bauxite particles to provide a nucleation point for hydration product formation. By increasing the availability of reactive silicon and calcium in AAMs, GGBFS promotes the formation of C-S-H gels, which can coexist with N-A-S-H gels or form hybridized C(N)-A-S-H gels, ultimately increasing the mechanical strength. The progressive addition of GGBFS results in the continuous generation of C-S-H and C-A-S-H gels, leading to pore filling between the precursor particles and a reduction in the porosity of the hardened paste.

In summary, the alkaline environment provided by the activator disrupts the silica–oxygen network on the GGBFS surface, thereby promoting the release of Ca^2+^, Si^4+^, and Al^3+^ ions into the solution. As [SiO_4_]^4−^ and [AlO_4_]^5−^ begin to dissolve from the BX component, the rapid release of Ca^2+^ from the GGBFS leads to the formation of localized high concentrations. This results in the precipitation of initial calcium-bearing phases, potentially including Ca(OH)_2_, particularly under the conditions of high-calcium GGBFS and insufficient reactive silica. These phases subsequently serve as nucleation sites for the formation of C-S-H and C-A-S-H gels. Ca(OH)_2_ crystals are precipitated and enriched around the GGBFS and bauxite particles, providing nucleation points for the formation of the hydration products. The released Ca^2+^, Si^3+^, and Al^3+^ ions then react with the alkaline activator and dissolve components from BX, leading to the formation of C-S-H and C-A-S-H gels. The presence of aluminum (Al) from BX is critical, as it becomes incorporated into the C-S-H structure, resulting in the formation of C-A-S-H. In terms of N-A-S-H/C-A-S-H interactions, C-A-S-H gels can coexist with N-A-S-H gels (primarily formed through BX activation), and even form hybrid C(N)-A-S-H gels.

### 4.2. Discussion of Insufficient Research Contributions

Although these findings provide a theoretical foundation for the high-value utilization of bauxite residue, facilitating its application in alkali-activated materials (AAMs), they also encourage the further development and optimization of industrial waste for AAM production. This contributes to the development of sustainable building materials and environmental protection. However, this study was conducted under controlled laboratory conditions, and the long-term performance and durability of AAMs in real-world environments require further investigation. This study employed a specific composite alkaline activator, without comparing it to other activators or activation systems. Different activators have varying effects on material properties. Therefore, future research will attempt to use organic bases, ionic liquids, or waste-derived alkalis as activators to assess their impact on material performance. Additionally, the study lacked microstructural analysis regarding the influence of activators on the performance of alkali-activated bauxite slag (AABG), resulting in a lack of microscopic validation of the macroscopic test results. A comparative microstructural analysis of AABGs under various research conditions would be beneficial for elucidating the microstructural evolution during the hydration process.

While the results of this study fill a gap in the application of bauxite residue-based materials in rapid hardening engineering, future research should focus on expanding production processes, evaluating environmental impacts, and exploring microstructural evolution to optimize material properties. Specifically, future studies should address the following aspects:

(1) Exploring simplified preparation methods for bauxite residue-based cementitious materials: Research should focus on low-energy, efficient activation methods, as well as the potential for combining bauxite residue with other solid wastes (e.g., spontaneous coal gangue and carbide slag) and adjusting their workability and performance. These improvements would facilitate large-scale production and processing, making these materials more suitable for use in construction operations and building industries.
(2) Investigating the mechanisms of microstructural evolution to optimize material performance: Understanding the microstructural evolution and strengthening mechanisms of cementitious materials is crucial for enhancing the performance of AAMs. This requires more accurate microstructural data analysis techniques (e.g., determining the new mineral phase content via X-ray diffraction (XRD), measuring the leaching ratio of precursor elements, and using nuclear magnetic resonance (NMR) to clarify polymerization degree changes) and the development of new research methods (e.g., machine learning for image recognition of inert material distribution, establishing specific hydration heat models, and compact packing models). Defining the pathways for microstructural enhancement is vital for improving performance.
(3) Further evaluating environmental impacts: Future research should focus on the carbon emissions associated with the entire lifecycle of AAM production, from the generation, transportation, and preparation of bauxite residue (and other raw materials) to the final AAM product. Carbon reduction rates and industrial waste recycling rates should be quantified. In addition, establishing energy and carbon emission data for the entire process, from precursor production to AAM preparation, is essential. The integration of industrial and biomass wastes, along with a circular economy model, will accelerate the transition of the industry toward greener, low-carbon practices.

## 5. Conclusions

This study aimed to utilize BX and GGBFS as precursors, employ composite alkali activators, and optimize mix ratios through response surface methodology to systematically investigate the effects of various parameters on the mechanical properties and hydration characteristics of BX-GGBFS alkali-activated composite materials. The effects of the alkaline activator content, sodium silicate modulus, water–solid ratio, and GGBFS content on AABG cementitious materials were investigated from macroscopic and microscopic perspectives. The primary conclusions derived from the experimental findings of this study are as follows:

(1) Based on the experimental data, a quadratic polynomial regression model was established to predict the 7 d and 28 d mechanical strengths of the BX-GGBFS alkali-activated cement material; variance analysis revealed that the model had a high coefficient of determination (*f*_3c_ = 0.9803, *f*_28c_ = 0.9789), and the model fitted well. The alkali content (*X*_1_) and water–solid ratio (*X*_3_) significantly affect the compressive strength, and the optimum mixture ratio is as follows: alkali content, 4%; sodium silicate modulus, 1.3; and water–solid ratio, 0.32. The error between the predicted and measured values was within the acceptable range.
(2) The enhancement of the mechanical properties of AABGs by GGBFS was more pronounced. As the percentage of GGBFS increased, the strength increased continuously. However, when the GGBFS content exceeded 35%, the strength rate increased, and the optimization effect appeared to decrease. Active Ca^2+^ ions from GGBFS rapidly participated in the reaction, promoting gel formation under alkaline conditions. The hydration product gels filled the pores between the precursor particles, and the improved particle size distribution refined the pore structure, thereby enhancing the material strength.
(3) With the incorporation of GGBFS, the fluidity and setting time of the AABG system decreased gradually. Although GGBFS improved the particle size distribution and enhanced the flowability, the presence of Ca^2+^ ions accelerated the depolymerization of precursor particles and the formation of hydration products (C-A-S-H and C-S-H gels), leading to rapid setting of the slurry.
(4) XRD, SEM–EDS, FTIR, and TG-DSC analyses demonstrated that the hydration products of AABG were predominantly C-S-H and C-(N)-A-S-H gels in the form of SiQ_3_ and SiQ_4_ tetrahedra, accompanied by a minor presence of analcime and gismondine. As the GGBFS doping level increased, the calcium-to-silicon (Ca/Si) ratio of the hydration product gradually decreased from 0.97 to 1.21, 0.7, and 1.0. Concurrently, the silicon-to-aluminum (Si/Al) ratio increased from 0.51 to 1.71, 0.64, and 1.27. This indicates an increase in the content of silicate structures and a more intricate gel network, contributing to enhanced mechanical properties.

In conclusion, the AABGs prepared from GGBFS had a GGBFS content of 35%, an alkali content of 4%, a sodium silicate modulus of 1.3, and a water–solid ratio of 0.32, and had good strength and workability. Increasing the Na_2_O equivalent content and decreasing the W/S ratio can optimize the properties of the AABGs.

These findings provide a theoretical foundation for the high value-added utilization of bauxite residue, promoting its application in AAMs. The results encourage further development and optimization of AAMs using industrial waste, contributing to the development of sustainable construction materials and environmental conservation. However, this study was conducted under controlled laboratory conditions, and the long-term performance and durability of AABG materials in real environments require further investigation. Future research should focus on scaling up the production process, evaluating the environmental impact, and exploring microstructural evolution to optimize material properties.

## Figures and Tables

**Table 1:** Chemical composition of raw materials (wt.%).

Materials	CaO	SiO_2_	Al_2_O_3_	MgO	SO_3_	Fe_2_O_3_	K_2_O	Na_2_O	LOI
BX	0.562	21.049	69.234	0.092	0.009	1.997	0.189	-	6.868
GGBFS	50.219	25.615	12.070	5.175	2.408	0.314	0.301	0.408	3.49

**Table 2:** Test factors and level design of RSM-CCD.

Independent Variable Factor	Code Level				
	−1.5 (−α)	−1 (Low Level)	0 (Center Level)	1 (High Level)	1.5 (α)
*X*_1_ (%)	1	2	4	6	7
*X*_2_ (M)	1	1.1	1.3	1.5	1.6
*X*_3_	0.29	0.30	0.32	0.34	0.35

**Table 3:** Experimental design of response surface for optimal ratio of binary bauxite powder cementing material.

Samples	m(GGBFS)/m(BX)	Alkaline Activators		W/C
		Modulus	Na_2_O (%)	
1	65:35	1	4	0.32
2	65:35	1.1	2	0.3
3	65:35	1.1	6	0.3
4	65:35	1.1	2	0.34
5	65:35	1.1	6	0.34
6	65:35	1.3	4	0.29
7	65:35	1.3	1	0.32
8	65:35	1.3	4	0.32
9	65:35	1.3	4	0.32
10	65:35	1.3	4	0.32
11	65:35	1.3	4	0.32
12	65:35	1.3	4	0.32
13	65:35	1.3	4	0.32
14	65:35	1.3	7	0.32
15	65:35	1.3	4	0.35
16	65:35	1.5	2	0.3
17	65:35	1.5	6	0.3
18	65:35	1.5	2	0.34
19	65:35	1.5	6	0.34
20	65:35	1.6	4	0.32

**Table 4:** Strength test results.

Samples	Compressive Strength (MPa)									
	1	2	3	4	5	6	7	8	9	10
*f*_3*c*_	54.85	45.34	69.13	19.52	62.59	70.94	9.34	49.83	52.84	52.75
*f*_28*c*_	81.56	70.33	98.42	41.85	92.72	103.56	30.82	78.64	82.19	82.08
**Samples**	**11**	**12**	**13**	**14**	**15**	**16**	**17**	**18**	**19**	**20**
*f*_3*c*_	57.75	50.91	56.18	57.25	40.83	37.67	53.36	16.51	49.63	48.25
*f*_28*c*_	82.08	79.91	86.13	87.42	70.01	67.28	85.81	44.33	91.43	79.77

**Table 5:** ANOVA test results.

Compiled Source	*f*_3c_		*f*_28c_	
	*F*_Value_	*p*_Value_	*F*_Value_	*p*_Value_
Model	55.19	<0.0001	51.66	<0.0001
*X*_1_	19.59	0.0013	1.66	0.2262
*X*_2_	283.38	<0.0001	297.55	<0.0001
*X*_3_	84.51	<0.0001	58.60	<0.0001
*X*_1_ *X*_2_	4.1	0.0704	1.57	0.2389
*X*_1_ *X*_3_	0.7	0.4215	2.51	0.1444
*X*_2_ *X*_3_	16.97	0.0021	23.28	0.0007
*X*_1_^2^	0.96	0.35	0.36	0.5645
*X*_2_^2^	85.49	<0.0001	76.80	<0.0001
*X*_3_^2^	0.97	0.349	2.97	0.1153
Lack of fit	1.14	0.4435	1.19	0.4257
*R* ^2^	0.9803		0.9789	
*R* ^2^_adj_	0.9625		0.9660	
*R* ^2^_pre_	0.9773		0.9777	
*C.V*	6.60%		4.88%	

**Table 6:** Response optimization standards.

Independent Variable	Low Level	High Level
*X*_1_ (%)	4	6
*X*_2_ (M)	1.1	1.3
*X*_3_	0.3	0.32

**Table 7:** Correlation parameters of AABG 28 d peak fitting under different GGBFS dosages.

	SiQ_0_	SiQ_1_	SiQ_2_	SiQ_3_	SiQ_4_	(SiQ_3_ + SiQ_4_)/(SiQ_1_ + SiQ_2_)
BG-1-28 d	8.124	27.101	26.488	26.731	11.557	0.714
BG-2-28 d	13.405	13.026	29.804	19.850	23.915	1.022
BG-3-28 d	12.757	24.652	15.752	26.493	20.346	1.159
BG-4-28 d	7.851	4.814	29.118	36.134	22.084	1.305

**Table 8:** Thermogravimetric mass loss table of AABGs with different GGBFS contents.

Temperature	BG-1	BG-2	BG-3
30–200 °C	10.102	6.443	4.566
200–600 °C	2.508	3.366	4.208
600–750 °C	0.968	0.579	0.245

**Table 9:** EDS spectral data of AABG with different GGBFS contents.

Point	C	O	Na	Mg	Al	Si	Ca
Point #1	15.79	42.55	3.18	18.32	18.54	9.48	9.23
Point #2	14.69	41.04	2.4	1.5	7.46	12.81	15.53
Point #3	11.55	40.17	2.26	1.51	21.82	14.34	8.36
Point #4	15.64	46.5	2.46	2.54	10.14	12.9	9.81
Point #5	13.7	42.06	2.27	2.4	19.01	12.16	10.4
Point #6	15.8	47.83	1.72	1.34	11.13	11.05	11.15

## Data Availability

The original contributions presented in this study are included in the article. Further inquiries can be directed to the corresponding author.
